# *Borrelia burgdorferi* membranes are the primary targets of reactive oxygen species

**DOI:** 10.1111/j.1365-2958.2008.06204.x

**Published:** 2008-05-01

**Authors:** Julie A Boylan, Kevin A Lawrence, Jennifer S Downey, Frank C Gherardini

**Affiliations:** 1 National Institute of Allergy and Infectious Diseases, Rocky Mountain Laboratories 903 S 4th Street, Hamilton, MT 59840, USA; 2 University of Southern California Los Angeles, CA, USA

## Abstract

Spirochetes living in an oxygen-rich environment or when challenged by host immune cells are exposed to reactive oxygen species (ROS). These species can harm/destroy cysteinyl residues, iron-sulphur clusters, DNA and polyunsaturated lipids, leading to inhibition of growth or cell death. Because *Borrelia burgdorferi* contains no intracellular iron, DNA is most likely not a major target for ROS via Fenton reaction. In support of this, growth of *B. burgdorferi* in the presence of 5 mM H_2_O_2_ had no effect on the DNA mutation rate (spontaneous coumermycin A1 resistance), and cells treated with 10 mM *t*-butyl hydroperoxide or 10 mM H_2_O_2_ show no increase in DNA damage. Unlike most bacteria, *B. burgdorferi* incorporates ROS-susceptible polyunsaturated fatty acids from the environment into their membranes. Analysis of lipoxidase-treated *B. burgdorferi* cells by Electron Microscopy showed significant irregularities indicative of membrane damage. Fatty acid analysis of cells treated with lipoxidase indicated that host-derived linoleic acid had been dramatically reduced (50-fold) in these cells, with a corresponding increase in the levels of malondialdehyde by-product (fourfold). These data suggest that *B. burgdorferi* membrane lipids are targets for attack by ROS encountered in the various stages of the infective cycle.

## Introduction

*Borrelia burgdorferi*, the causative agent of Lyme disease, survives and proliferates in distinctly different niches, including its arthropod vector and various mammalian hosts. These ‘micro’ environments provide their own distinct sets of advantages and challenges to *B. burgdorferi*. For example, during the initial stages of infection of the mammalian host, immune cells attempt to prevent *B. burgdorferi* from establishing an infection using several systems including those generating bacteriocidal reactive oxygen species (ROS) [e.g. superoxide radicals (O_2_^-^), hydrogen peroxide (H_2_O_2_) and hydroxyl radicals (OH)] and reactive nitrogen species (RNS) [e.g. nitric oxide (NO) and peroxynitrite] (Storz and Imlay, 1999). In order for *B. burgdorferi* to successfully colonize a new host and cause disease, they must overcome the challenges posed by the innate immune system including the deleterious effects of ROS/RNS compounds.

The effects of ROS/RNS on cells have been extensively investigated. These highly reactive compounds have been shown to damage cellular macromolecules including DNA, proteins and cellular membranes. The damage to membranes can arise through either lipid or membrane protein damage. In eukaryotes, membrane lipids are a major target of ROS. Free radicals attack polyunsaturated fatty acids in membranes and initiate lipid peroxidation. A primary effect of this is a decrease in membrane fluidity which affects the physical properties of the membrane altering the function of membrane-associated proteins. Once lipid peroxides form, they react with adjacent polyunsaturated lipids causing an amplification of the damage. Lipid peroxides undergo further oxidation to a variety of products, including aldehydes, which subsequently react with and damage membrane proteins. However, in bacteria, it is assumed that lipids are not subject to the oxidative damage observed in eukaryotic cells. Only certain polyunsaturated lipids, such as linoleic acid and linolenic acid, are susceptible to oxidation (Gutteridge and Halliwell, 1990), and it is clear that most bacteria do not synthesize or incorporate these types of lipids in their cell membrane. Two notable exceptions are the photosynthetic bacteria which synthesize and incorporate significant levels of linoleic acid in their membrane (Tasaka *et al*., 1996) and *Helicobacter pylori* membranes which contain between 0.5% and 3% linoleic acid (Ursini and Bindoli, 1987).

Instead, it has been shown that the most damaging effects of ROS in bacteria result from the interactions of H_2_O_2_ with ‘free’ Fe^2+^ (Imlay, 2003), generating very reactive OH (Fenton reaction). Because of this reactivity, the effect on any given biomolecule will depend largely upon proximity to the target. Because Fe^2+^ localizes along the phosphodiester backbone of nucleic acid, DNA is a major target of OH. This reactive species can pull electrons from either the base or sugar moieties, producing a variety of lesions including single- and double-stranded breaks in the backbone and chemical cross-links to other molecules. These strand breaks and other lesions block DNA replication and contribute to OH toxicity and cell death. Other base damage, which does not hinder replication, may result in a significant increase in mutation rates.

The intracellular biochemistry of *B. burgdorferi* suggests that the primary cellular target of ROS may be distinct from that described in other bacteria such as *Escherichia coli*. In *E. coli*, the extent of DNA damage due to H_2_O_2_ and Fenton chemistry is directly proportional to Fe metabolism and the free Fe concentration within the cell (10 µM) (Keyer and Imlay, 1996). As the intracellular Fe concentrations of *B. burgdorferi* are estimated to be < 10 atoms per cell (Posey and Gherardini, 2000), it seems unlikely that DNA is a primary target for ROS. Therefore, the purpose of this study is to determine the biochemical effects of ROS on *B. burgdorferi*, including growth effects and biological/physical damage.

## Results

### Effect of ROS on *B. burgdorferi* cells

**Fig. 1:**
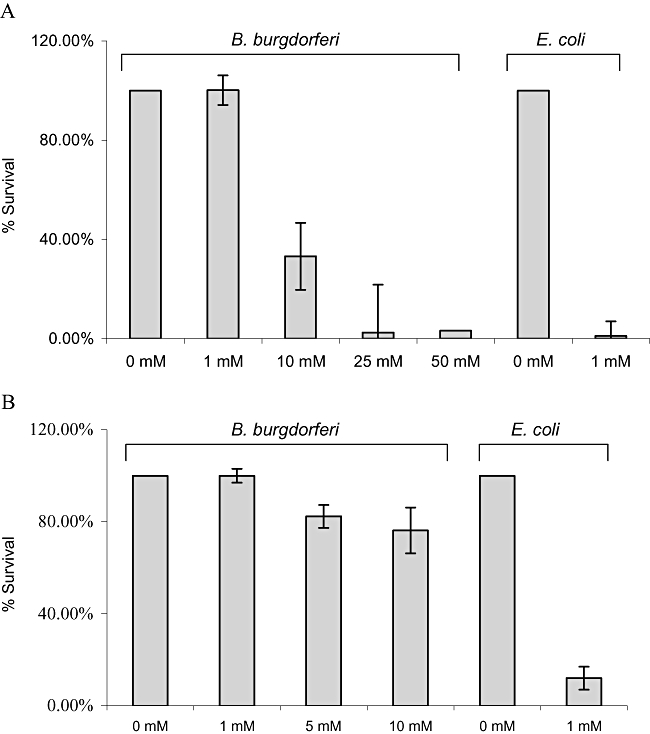
*B. burgdorferi* is highly resistant to treatment with both *t*-butyl hydroperoxide and H_2_O_2_. Microaerobic cultures of *B. burgdorferi* strain B31A3 were grown to a cell density of 5 × 10^7^ cells ml^−1^ and treated with varying concentrations of (A) *t*-butyl hydroperoxide (0–50 mM) and (B) H_2_O_2_ (0–50 mM). The number of surviving cells was determined by plating.

In order to determine what the cellular targets of ROS in *B. burgdorferi* are, we first needed to determine the sensitivity of *Borrelia* cells to various oxidants. Microaerobic cultures of *B. burgdorferi* strain B31A3 were grown to a cell density of 5 × 10^7^ cells ml^−1^, treated with varying concentrations of H_2_O_2_ (0–50 mM) or *t*-butyl hydroperoxide (0–50 mM) and the number of surviving cells determined by plating. The results are shown in Fig. 1. When cells were exposed to 10 mM *t*-butyl hydroperoxide, approximately 50% of the cells survive (Fig. 1A). In contrast, when *E. coli* cells were exposed to 1 mM *t*-butyl hydroperoxide, approximately 1% of the cells survive. *B. burgdorferi* cells were much more resistant to *t*-butyl hydroperoxide than *E. coli* cells, with a survival rate of approximately 100% at 1 mM *t*-butyl hydroperoxide. This trend was observed when cells were exposed to H_2_O_2_. *E. coli* cells exposed to 1 mM H_2_O_2_ have approximately 10% survivability, while 100% of the *B. burgdorferi* cells survive at this concentration, and approximately 80% survive when exposed to 10 mM H_2_O_2_ (Fig. 1B). Similar results were obtained when cells were exposed in HN (Hepes-NaCl) buffer, suggesting that the high-level resistance to ROS was not due to the interaction of ROS with components of Barbour-Stoenner-Kelly (BSK-II) growth medium. Taken together, these data indicated that *B. burgdorferi* strain B31A3 was highly resistant to exposure to both *t*-butyl peroxide and H_2_O_2_.

### *Borrelia burgdorferi* DNA is not the primary target of ROS

For most bacteria, DNA is the major target of ROS causing a wide variety of DNA lesions. This is in part due to the localization of ‘free’ Fe^2+^ along the phosphodiester backbone of nucleic acids, putting the DNA in close proximity to the active species formed via the Fenton reaction. However, *B. burgdorferi* has been shown to harbour few genes encoding orthologues of known iron-containing proteins, does not require Fe for growth and has intracellular Fe concentrations estimated to be < 10 atoms per cell (Posey and Gherardini, 2000). Taken together, these observations strongly suggest that *B. burgdorferi* DNA is not a major target for ROS. To test this hypothesis, different techniques were used to measure DNA damage in *B. burgdorferi* cells after exposure to ROS.

**Fig. 2:**
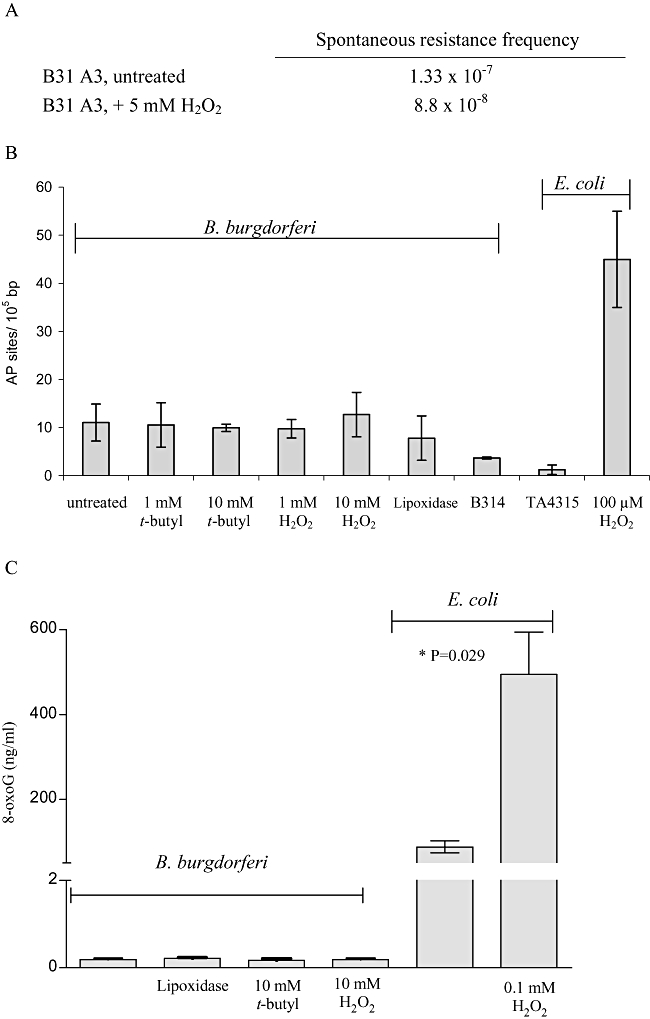
The addition of oxidants does not damage *Borrelia* DNA. A. *B. burgdorferi* B31A3 cells were grown under microaerobic conditions to a cell density of 5 × 10^7^ cells ml^−1^, treated with 5 mM H_2_O_2_ and plated in the presence and absence of 250 ng ml^−1^ coumermycin A_1_. The resistance frequency was calculated as the number of colonies that are Cou^R^ per total number of cells plated. B. *B. burgdorferi* B31A3 and B314 cells were grown in BSK-II to a cell density of 5 × 10^7^ cells ml^−1^, treated with various oxidants for 1 h, and DNA isolated. The DNA was then mixed with an aldehyde reactive probe (Oxford Biomedical Research) labelled with biotin and detected with an HRP-streptavidin conjugate. The colour development was monitored at 450 nm. The number of aldehyde reactive probe (DNA base lesions) per 10^5^ bp DNA was determined using a standard curve. *E. coli* TA4315 (*ahpCF*) (Storz *et al*., 1989) cells were grown in minimal media to OD_600_ of 0.4, treated with 100 µM H_2_O_2_ for 30 min, and DNA isolated. The number of base lesions was determined as described above. C. *B. burgdorferi* B31A3 cells were grown in BSK-II to a cell density of 5 × 10^7^ cells ml^−1^, treated with various oxidants for 1 h, and DNA isolated. The DNA was then converted to single-stranded and digested with P1. The resulting mixture was used in an 8-oxoguanine ELISA assay (Oxford Biomedical Research). The concentration of 8-oxoguanine was determined using a standard curve. *E. coli* CM1319 (*mutM*) cells were grown in LB to OD_600_ of 0.4, treated with 100 µM H_2_O_2_ for 1 h and DNA isolated. The concentration of 8-oxoguanine was determined as above.

One reliable indicator of DNA damage by ROS in a cell is an increase in the spontaneous mutation rate. In *B. burgdorferi*, mutations that confer resistance to coumermycin A1, which targets the β subunit of DNA gyrase, have been mapped to *gyrB*, the gene encoding DNA gyrase B (Samuels *et al*., 1994). In each case, a single point mutation correlated with this drug resistance. To determine if exposure to oxidants increases DNA damage by increasing point mutations, *B. burgdorferi* B31A3 cells grown under microaerobic conditions were treated with 5 mM H_2_O_2_ and plated in the presence and absence of 250 ng ml^−1^ coumermycin A1. The mutation frequency was calculated as the number of colonies that are Cou^R^ per total number of cells plated. The spontaneous resistance frequency of treated cells was approximately equivalent to that of untreated cells, 8.8 × 10^−8^ and 1.33 × 10^−7^, respectively, indicating no increase in the number of point mutations (Fig. 2A). Also, no increase in point mutations was observed when cells were treated with higher concentrations of H_2_O_2_ (10 mM) or when treated with *t*-butyl hydroperoxide (5 and 10 mM) (data not shown).

Another effective way to determine DNA damage is by measuring the number of apurinic/apyrimidinic sites (AP). AP sites in DNA, where the DNA base is lost, can be generated spontaneously under physiological conditions by hydrolysis of the N-glycosylic bond, or can also be formed by DNA-damaging agents (Lindahl and Nyberg, 1972), such as UV, alkylating agents or OH. They are also intermediates in the base excision repair pathway (BER) (Weiss and Grossman, 1987; Friedberg and Hanawalt, 1988; Wallace, 1988). Thus, the cellular steady state level of AP sites would increase as a consequence of base modifications and their subsequent repair. As AP sites are bypassed inefficiently by DNA polymerase in bacterial cells, DNA lesions can result in a significant block in DNA replication. Therefore, the number of AP sites can serve as a sensitive indicator of DNA damage resulting from oxidative stress (Kubo *et al*., 1992). To determine whether ROS can damage *B. burgdorferi* DNA, strain B31A3 cells grown under microaerobic conditions were treated with H_2_O_2_ (1 or 10 mM), *t*-butyl hydroperoxide (1 or 10 mM) or lipoxidase (an enzyme which specifically catalyses the hydroperoxidation of lipids containing a *cis*, *cis*-pentadiene structure, such as linoleic acid). The DNA was isolated and assayed for AP sites. The results are shown in Fig. 2B. In all cases, the numbers of AP sites per 10^5^ bp DNA were equivalent, indicating that the addition of oxidants did not increase the number of DNA base lesions. In contrast, when *E. coli* strain TA4315 cells (*ahpCF*) (Storz *et al*., 1989) were treated with 100 µM H_2_O_2_, the number of AP sites increased ∼10-fold over untreated cells. These data suggest that *B. burgdorferi* DNA was not a major target for oxidative damage under these conditions.

It is of note to mention that the total number of AP sites per 10^5^ bp DNA is approximately 10-fold higher in untreated *Borrelia* B31A3 DNA than in untreated *E. coli* DNA. The *Borrelia* genome consists of a linear chromosome and multiple linear and circular plasmids. Numerous observations indicate that functional telomeres require interaction with DNA damage repair proteins, suggesting that the DNA damage repair machinery, including the BER, is involved in replication of telomeres and protection of functional chromosome ends (Verdun and Karlseder, 2007). An important intermediate in BER is the apurinic or abasic site. Therefore, it was possible that the higher numbers of AP sites in untreated *Borrelia* DNA was due to the number of telomeres present in the genome. The *B. burgdorferi* strain B31A3 harbours 11 linear plasmids and a linear chromosome with ∼100 unpaired bases in the telomere loops per genome or 10 bases per 10^5^ bp DNA (Hinnebusch and Barbour, 1991; Chaconas, 2005). Untreated *Borrelia* B31A3 contains 11 ± 3.8 AP sites per 10^5^ bp DNA, suggesting that the elevated number of AP sites in *Borrelia* was due to the number of telomeres. To test this hypothesis, the AP sites were measured in DNA isolated from untreated *B. burgdorferi* strain B314 which harbours no linear plasmids (Sadziene *et al*., 1993). The number of unpaired bases in the telomere loops per genome was estimated to be 10, which corresponds to 1 base per 10^5^ bp DNA, and only 3.6 ± 0.2 AP sites per 10^5^ bp DNA were detected, supporting the hypothesis that the high number of AP sites in untreated *Borrelia* DNA was due to the number of unpaired bases in the telomeres. The analyses of the AP sites strongly suggested that *B. burgdorferi* DNA was not the major target for oxidative damage.

In addition to the generation of abasic sites, oxygen radicals often damage DNA through the formation of 8-oxoguanine lesions (Nakamura *et al*., 2000). 8-Oxoguanine, through its ability to mispair with bases other than cytosine, likely plays a role in DNA mutagenesis. Consequently, 8-oxoguanine is often used as a marker of oxidized DNA damage. To determine whether ROS can damage *B. burgdorferi* DNA and cause 8-oxoguanine lesions, strain B31A3 cells grown under microaerobic conditions were treated with H_2_O_2_ (5 or 10 mM), *t*-butyl hydroperoxide (5 or 10 mM) or lipoxidase. The DNA was isolated and assayed for 8-oxoguanine using an Enzyme-Linked ImmunoSorbent Assay. The results are shown in Fig. 2C. In all cases, the amount of 8-oxoguanine in *Borrelia* cells was below the detection limit of the assay. In contrast, when a MutM (the specific glycosylase that removes the 8-oxoguanine)-deficient *E. coli* strain was treated with 100 µM H_2_O_2_, the amount of 8-oxoguanine sites increased ∼fivefold over untreated cells. It is important to point out that no MutM homologue has been identified in the genome of *B. burgdorferi.* Taken together, these data suggested that *B. burgdorferi* DNA was not a major target for oxidative damage under these conditions.

### The membranes of *B. burgdorferi* are targeted during oxidative stress

In eukaryotes, membrane lipids are a major target of ROS. Free radicals can attack polyunsaturated fatty acids, such as linoleic acid and linolenic acid, in membranes and initiate lipid peroxidation. This reaction can cascade throughout the membrane to adjacent polyunsaturated fatty acids, decreasing membrane fluidity and generating more toxic products such as aldehydes (Imlay, 2003). Because most bacterial membranes contain saturated and monounsaturated fatty acids rather than ‘reactive’ polyunsaturated lipids, peroxidation of lipids in bacterial membranes is not considered a problem. As *B. burgdorferi* cannot synthesize their own lipids, they must instead scavenge them. Therefore, it seems likely that their membrane composition would reflect the host's lipid profile or that of their growth medium (Barbour, 1984; Fraser *et al*., 1997) and would contain some polyunsaturated fatty acids. To determine if *B. burgdorferi* contains polyunsaturated lipids, *B. burgdorferi* strain B31A3 was grown under anaerobic conditions and analysed for fatty acid composition by Lipid Technologies (Austin, MN). The results are shown in Table 1 and are reported as percentage of total fatty acid content. Linoleic acid comprised ∼10% of the total lipid content and the linolenic acid content was measured to be ∼1%, indicating that *Borrelia* cells do contain lipids that are susceptible to ROS damage. The amount of linoleic acid and linolenic acid present in *Borrelia* reflected the amount present in the media. Therefore, these results suggested that the amount of these fatty acids in the membranes would vary as availability varies.

**Table 1:** Fatty acid composition of *B. burgdorferi* grown in BSK-II.

Fatty acid	Whole cells^a^
Butyric acid (c4:0)	0.0
Caprylic acid (c8:0)	0.0
Lauric acid (c12:0)	0.0
Myristic acid (c14:0)	2.4
Pentadecanoic (c15:0)	1.3
Palmitic acid (c16:0)	54.7
Palmitoleic acid (c16:1n7)	1.9
Margaric acid (c17:0)	1.0
Stearic acid (c18:0)	4.6
Oleic acid (c18:1n9)	21.9
Linoleic acid (c18:2n6)	11.6
α-Linolenic acid (c18:3n3)	0.6
Behenic acid (c22:0)	0.0
Lignoceric acid (c24:0)	0.0

a Calculated as percentage of total fatty acid.

To determine if *Borrelia* polyunsaturated lipids can undergo lipid peroxidation, *B. burgdorferi* B31A3 cells grown microaerobically were treated with 1 mM *t*-butyl hydroperoxide or 250 mg of lipoxidase, and the cell pellets were analysed for fatty acid composition (Industrial Laboratory). The results are shown in Table 2 and are reported as the percentage of total cell mass. The per cent of linoleic acid in the total cell mass decreased with treatment, while the levels of oleic acid (c18:1n9) and pentadecanoic acid (c15:0) remained relatively constant. Untreated cells contained 0.04% linoleic acid, while cells treated with *t*-butyl hydroperoxide contained 10-fold less (0.004%) linoleic acid and cells treated with lipoxidase had no detectable linoleic acid present in the sample. These data indicated that the linoleic acid present in *B. burgdorferi* membranes can be oxidized by ROS.

**Table 2:** Effects of ROS and lipoxidase on the composition of fatty acids in *B. burgdorferi*.

Fatty acids	Untreated^a^	*t*-Butyl hydroperoxidea	Lipoxidasea
Pentadecanoic (c15:0)	0.0344	0.0332	0.0262
Oleic acid (c18:1n9)	0.3264	0.3165	0.2469
Linoleic acid (c18:2n6)	0.0393	0.0044	< 0.001

a Calculated as percentage total cell mass.

**Fig. 3:**
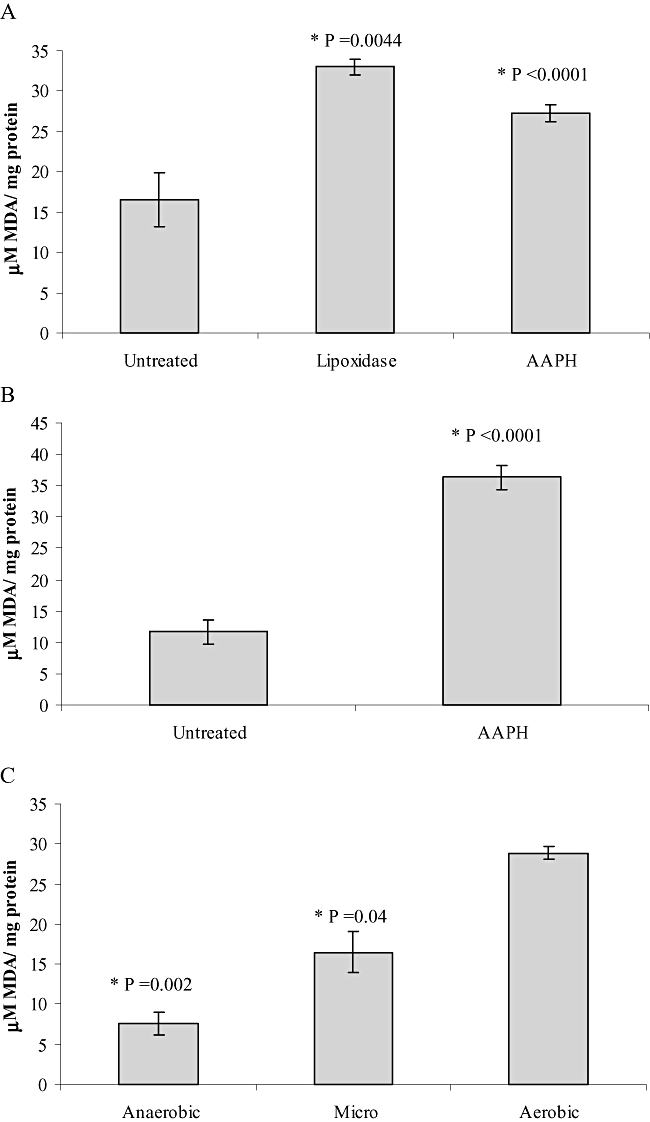
The amount of the lipid peroxide intermediate MDA increases with increasing concentrations of O_2_ and with exposure to oxidants. A. *B. burgdorferi* strain B31A3 was grown under microaerobic conditions, the culture split and treated with either 5 mM AAPH or 250 mg of lipoxidase for 4 h at 34°C. To measure the amount of MDA, the cells were derivatized with thiobarbituric acid and analysed by HPLC. MDA standards were prepared in methanol for comparison. The calculated *P*-values indicated that the two treated samples were significantly different from the untreated sample. B. Mouse myeloma SP2/O cells were cultured with HYQ-CCM1 (HyClone) medium at 37°C in a humidified 5% CO_2_ atmosphere, treated with 1 mM AAPH at 37°C for 4 h (Chotimarkorn *et al*., 2005) and MDA measured as above. The calculated *P*-value indicated that the treated sample was significantly different from the untreated sample. C. *B. burgdorferi* strain B31A3 was grown aerobically, microaerobically and anaerobically to a cell density of 5 × 10^7^ cells ml^−1^ and MDA measured as above. The calculated *P*-values indicated that both the anaerobic and microaerobic samples were significantly different from the aerobic sample.

Malondialdehyde (MDA) is generated as a relatively stable end-product from the oxidative degradation of polyunsaturated fatty acids. MDA has thus been used as an indicator of lipid peroxidation (Gutteridge and Halliwell, 1990; Esterbauer *et al*., 1991). To further demonstrate that *Borrelia* lipids can undergo lipid peroxidation, B31A3 cells grown microaerobically were treated with 5 mM AAPH (free radical generator) or 250 mg of lipoxidase and MDA measured (Seljeskog *et al*., 2006). The results are shown in Fig. 3. Untreated cells contained ∼16.5 µM of MDA per mg of protein. When the cells were treated with AAPH or lipoxidase, the amount of MDA increased ∼1.5-fold (27.3 µM of MDA per mg of protein) and approximately twofold (33 µM of MDA per mg of protein) respectively (Fig. 3A). As a control, eukaryotic cells (mouse myeloma cells SP2) were treated with AAPH and MDA measured (Fig. 3B). Mouse myeloma cells have been used as a model system for the determination of phospholipid hydroperoxides (Chotimarkorn *et al*., 2005). In this case, the amount of MDA increased approximately threefold (36.3 µM of MDA per mg of protein) in the treated cells versus untreated cells (11.7 µM of MDA per mg of protein). Taken together, these data suggested that, like eukaryotic membranes, *Borrelia* membrane lipids were capable of undergoing lipid peroxidation.

As shown in Fig. 3A, there is a measurable quantity of MDA present even in untreated cells, suggesting that the membrane lipids are damaged without the addition of exogenous ROS. Oxidative damage is an unavoidable by-product of growth in an oxygen environment because superoxide anions and H_2_O_2_ are formed whenever molecular oxygen chemically oxidizes electron carriers. To determine if the *Borrelia* lipids are damaged from growth in an oxygen environment, *B. burgdorferi* B31A3 cells were grown under anaerobic, microaerobic and aerobic conditions and MDA measured. Figure 3C demonstrates that as the oxygen concentration increased, the amount of MDA increased. Cells grown under anaerobic conditions contained the lowest amount of MDA (7.6 µM of MDA per mg of protein), approximately twofold less than the measured amount in microaerobic cells (16.5 µM of MDA per mg of protein). Aerobically grown cells contained the highest amount of MDA, ∼1.5-fold greater than that observed in microaerobic cells (28.8 µM of MDA per mg of protein) and ∼3.7-fold greater than that observed in anaerobically grown cells. These data suggested that *Borrelia* lipids can be damaged during aerobic growth.

**Fig. 4:**
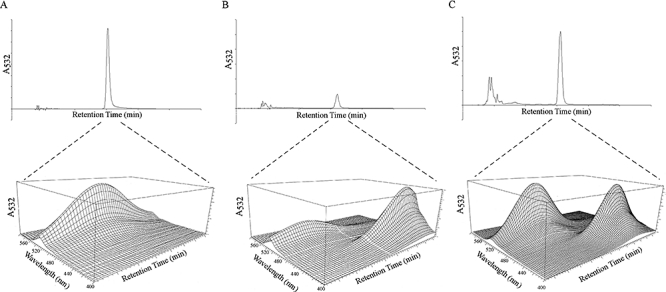
HPLC chromatograms with three corresponding dimensional diode array spectra scanned during elution of the peak with a retention time corresponding to the MDA standard. A. TBA-treated, authentic MDA standard with an absorbance maximum of 532 nm. Upper section, HPLC chromatogram; lower section, 3D diode array spectra. B. TBA-treated sample from *B. burgdorferi* strain B31A3 grown under anaerobic conditions. Upper section, HPLC chromatogram; lower section, 3D diode array spectra. C. TBA-treated sample from *B. burgdorferi* strain B31A3 grown under microaerobic conditions. Upper section, HPLC chromatogram; lower section, 3D diode array spectra.

Our High Performance Liquid Chromatography analyses of the MDA present in anaerobically grown *B. burgdorferi* cells showed a small peak with a retention time similar to that of the MDA standard. This was puzzling as little or no lipid peroxidation should occur under these conditions. To further characterize this ‘MDA’ peak, a three-dimensional diode array spectra was generated by scanning each sample during the elution of the peak (Fig. 4, lower sections). An authentic MDA standard was also scanned as a control (Fig. 4A, lower section). In the anaerobically grown cells, the spectrum shows that two compounds with different absorbance maximums (Fig. 4B, lower section) comprised the single retention time peak from the HPLC chromatogram (Fig. 4B, upper section). Based on this spectrum, the amount of actual MDA contributed < 15% of the total amount of material detected in the HPLC peak while the second contaminating peak contributed > 85%. Therefore, the amount of MDA in untreated anaerobically grown cells was considerably less than the 7.6 µM of MDA per mg of protein actually measured. However, in cells grown under microaerobic conditions, the MDA peak contributed more to the overall retention time peak when compared with the anaerobic spectrum, while the second contaminating peak stays relatively constant (Fig. 4C, lower section). These spectra demonstrated that the increase in the MDA retention time peak between anaerobically and aerobically grown cells was due to the increase in the amount of authentic MDA present.

**Fig. 5:**
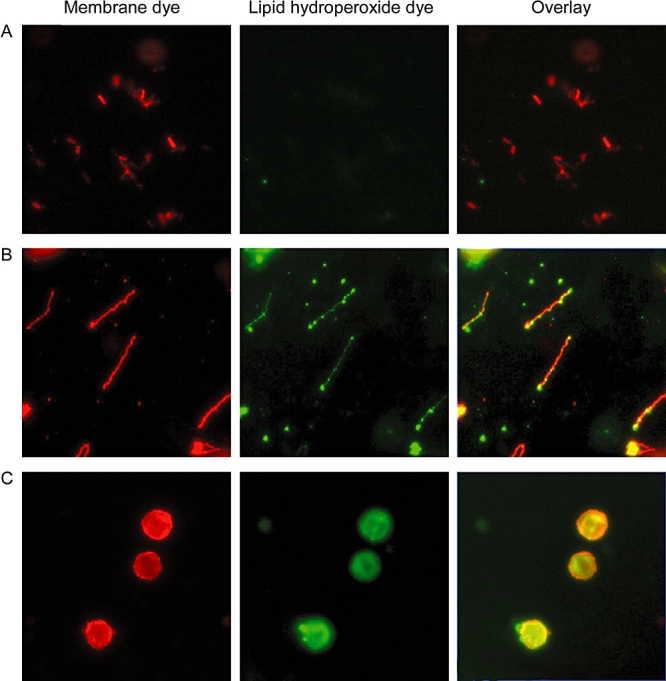
*Borrelia* cells contain lipid peroxides. A. *E. coli* Top10 cells were grown to OD_600_ of 0.4 and cells harvested. The whole cells were visualized with PKH26 Red Fluorescent Cell Linker Dye and the lipid hydroperoxides in the cell membrane were counterstained with DPPP. B. *B. burgdorferi* strain B31A3 was grown microaerobically to a cell density of 5 × 10^7^ cells ml^−1^ and stained with PKH26 and DPPP as described above. C. Mouse myeloma SP2/O cells were cultured with HYQ-CCM1 (HyClone) medium at 37°C in a humidified 5% CO_2_ atmosphere and stained with PKH22 and DPPP as described above.

The fluorescent probe diphenyl-1-pyrenylphophine (DPPP) has been used for detection of lipid hydroperoxides in cell membranes (Okimoto *et al*., 2000; Takahashi *et al*., 2001). In this method, the hydroperoxides are reduced with DPPP, resulting in the formation of the fluorescent arylphosphine oxide. To visualize the lipid hydroperoxides, *B. burgdorferi* B31A3 cells were labelled with DPPP and observed by fluorescence microscopy (Fig. 5B). *E. coli* Top10 cells and mouse myeloma SP2 cells were also labelled and visualized to serve as negative and positive controls respectively (Fig. 5A and C). Red Fluorescent dye was used to visualize the cell membranes. A strong fluorescence of DPPP was observed for B31A3 cells (Fig. 5B) and for the mouse myeloma cells (Fig. 5C), but not for the *E. coli* cells (Fig. 5A). An overlay of the two dyes demonstrates that the DPPP fluorescence of both the *Borrelia* and myeloma cells corresponds to the areas of red fluorescence. This indicated that lipid hydroperoxides were present on the *Borrelia* cell membranes and suggested that the membranes were damaged.

**Fig. 6:**
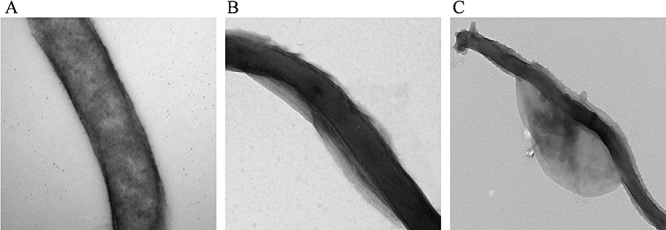
Treatment of *B. burgdorferi* cells with lipoxidase causes membrane damage. *B. burgdorferi* B31A3 cells were grown (A) anaerobically or (B) microaerobically to a cell density of ∼5 × 10^7^ cells ml^−1^. The microaerobic culture was split and one half exposed to (C) lipoxidase for 4 h. The cells were fixed with Karnovsky's phosphate and stained with 1% ammonium molybdate. Samples were examined and photographed using a Hitachi electron microscope.

To further demonstrate *Borrelia* membrane damage, *B. burgdorferi* B31A3 cells were grown under anaerobic and microaerobic conditions and visualized by negative stain using an electron microscope. Additionally, cells grown under microaerobic conditions were treated with 250 mg of lipoxidase and visualized by Electron Microscopy. Intact membranes were observed in cultures of B31A3 grown under anaerobic and microaerobic conditions (Fig. 6A and B respectively). However, in cultures treated with lipoxidase (Fig. 6C), a significant number of membrane blebs were seen surrounding the spirochetes, indicating membrane damage. Taken together, these data indicated that *Borrelia* membranes were a target for oxidative stress.

## Discussion

Most bacterial pathogens are faced with the challenge of overcoming ROS generated by the host immune system. These radicals can cause a great deal of damage to biological molecules both *in vitro* and *in vivo*. The potential cellular targets for ROS damage in bacteria include DNA, RNA, proteins and lipids, and extensive work has been done to determine the cellular targets of ROS that affect bacterial survival. To date, the most definitive work has been done on *E. coli*. It seems clear from several studies that the most physiologically relevant target of ROS in *E. coli* is DNA. The exposure to µM concentrations of ROS (i.e. H_2_O_2_) is sufficient to cause DNA damage, inhibit DNA replication, increase the mutation rate and often lead to cell death. This process leading to DNA damage begins with formation of O_2_^-^ and H_2_O_2_ from the oxidation of flavoproteins and/or the diffusion of these reactive species from the extracellular milieu. The subsequent oxidation of Fe-S proteins by ROS (e.g. O_2_^-^) leads to an increase in intracellular ‘free’ Fe^2+^ which triggers the reduction of H_2_O_2_ to OH (Fenton reaction). The highly reactive nature of OH limits its diffusion so that it generally reacts with molecules in close proximity to its origin (e.g. the Fe^2+^ associated with DNA). In DNA, OH oxidizes sugar and base moieties, producing radicals which ultimately generate lesions, including base modifications, strand breaks and chemical cross-links to other molecules. Base modifications lead to mismatching and increased mutation frequencies while more severe damage, such as strand breaks, prevents DNA replication, contributing to OH toxicity and cell death. Critical to this chemical process *in vivo* is the presence of iron. No other metal or non-metal electron carrier appears to be able to univalently reduce H_2_O_2_ to OH *in vivo* (Macomber *et al*., 2007).

These observations are critical in beginning to understand the oxidative damage/targets in *B. burgdorferi.* It has been shown in *E. coli* that the free-iron pool size determines the rate of oxidative DNA damage. For example, in wild-type *E. coli*, free iron levels are estimated to be 10 µM, yet H_2_O_2_ is only mildly genotoxic (Keyer and Imlay, 1996). However, in *E. coli* Fur^-^ mutants, intracellular iron concentration increases eightfold while survival is 10-fold lower when cells are exposed to H_2_O_2_. Because *B. burgdorferi* cells do not contain detectable levels of intracellular Fe, it seems unlikely that DNA is a major target for damage via the Fenton reaction in this bacterium (Posey and Gherardini, 2000). The experimental data present in this report suggested that this was the case. When *B. burgdorferi* cells were exposed to high concentrations of ROS (e.g. H_2_O_2_), there was no effect on the spontaneous mutation rate (Fig. 2A) or the number of DNA lesions (AP sites or 8-oxoguanine) (Fig. 2B and C). It should be noted that in these experiments, a wild-type strain of *B. burgdorferi* was used and presumably all of the endogenous oxidative stress enzymes were expressed. Therefore, it is possible that no DNA damage was observed because these enzymes are capable of detoxifying the cell and protecting nucleic acids from oxidation via the Fenton reaction. However, we do not believe this to be the case as the concentration of oxidants used in these experiments were significantly higher than concentrations known to cause DNA damage in *E. coli* and other pathogenic bacteria.

The lack of detectable DNA damage in *B. burgdorferi* cells under the conditions tested could be the result of very efficient DNA repair systems. Bacteria, such as *E. coli*, harbour genes encoding repair enzymes (i.e. MutM, MutY, Ung, AlkA, MutS, MutL, ExoIII, EndoVIII, PolI, RecJ). Key enzymes in the repair of 8-oxoguanine lesions resulting from the oxidation of DNA are the bifunctional glycosylases, such as MutM, MutY or EndoVIII. The first activity of these enzymes is to remove oxidized or ring-saturated bases while the second activity is to remove the resulting deoxyribose residue, generating a 3′-phosphate end (Krwawicz *et al*., 2007). This 3′-P is converted to a 3′-OH by various enzymes/pathways and the lesion is repaired by enzymes (e.g. ExoIII, PolI, LigI etc.) in the short or long BER pathways (S-BER, L-BER). Interestingly, the *B. burgdorferi* genome does not contain genes encoding homologues of MutM, MutY or EndoVIII, suggesting that it would be difficult for the cells to efficiently repair 8-oxoguanine sites using this pathway. *B. burgdorferi* does harbour the genes encoding the enzymes Ung (monofunctional gylcosylase, BB0053), MutS (BB0797, BB0098), MutL (BB0211), ExoIII (BB0114), PolI (BB0548), LigI (BB0552) and RecJ (BB0254) (Fraser *et al*., 1997) which are involved in excision and repair, via S-BER or L-BER, of deaminated, alkylated, methylated or mismatched bases. Clearly, these repair systems in *B. burgdorferi* do not seem as robust at defending against oxidation of DNA as those described in *E. coli.* The lack of key repair enzymes in this system may indicate that *B. burgdorferi* DNA is not subjected to the same challenge from ROS as is *E. coli.*

In most bacteria, ROS-mediated damage to lipids (lipid peroxidation) is very unlikely because of the lack of polyunsaturated fatty acids (e.g. linoleic acid) (Imlay, 2003). When it does occur, lipid peroxidation is initiated by the attack of free radicals on polyunsaturated fatty acids which decreases the membrane fluidity and**,** if these reactions propagate, lipid peroxides and their degradation products (e.g. aldehydes) in turn could damage proteins (Gutteridge and Halliwell, 1990). This would dramatically affect the function of transmembrane proteins and membrane-bound lipoproteins involved in the maintenance of membrane potential and solute transport, decreasing cell survivability. In contrast to most bacteria, *B. burgdorferi* membranes contained significant levels of unsaturated fatty acids, such as linoleic acid and linolenic acid, which were derived from the growth media (Table 1). Thus, it seemed possible that lipids and/or proteins rather than DNA are the primary targets of ROS in *B. burgdorferi.* When *Borrelia* cells are treated with oxidants, the levels of linoleic acid decreased while other fatty acids remain unaffected (Table 2). In addition, HPLC-based assays demonstrated that, as linoleic acid concentrations decrease, MDA (a toxic lipid peroxide intermediate) increased (Fig. 3). When these cells were examined by electron microscopy, damage to the membranes (membrane ‘blebs’) was observed (Fig. 6). These data indicated that unlike most other bacteria, *Borrelia* membranes were damaged by oxygen radicals.

As *B. burgdorferi* may be exposed to potentially harmful oxygen species at different stages of the infective cycle, the ability to protect its membrane lipids from ROS should be required for survival in the different host environments. In eukaryotic cells, where lipid peroxidation is a major consequence of oxidative attack, proteins which protect membranes have been well studied. For example, phospholipid hydroperoxide glutathione peroxidase, a member of the glutathione peroxidase family, has been identified in a variety of higher organisms. These enzymes are capable of reducing an assortment of hydroperoxy lipids, including oxidized phospholipids and cholesterol esters (Ursini and Bindoli, 1987), and protect complex membranes from oxidative damage. Much less is known about the enzyme(s) responsible for protecting unsaturated lipids from ROS in prokaryotes. In *H. pylori*, lipid hydroperoxide levels in *ahpC* mutants are approximately three times higher than in wild-type cells, suggesting a role for AhpC in reducing organic peroxides (Wang *et al*., 2006a,b). In addition, purified AhpC has been shown to reduce linoleic acid hydroperoxide *in vitro* (Baker *et al*., 2001). Because these antioxidant enzymes promote the *in vivo* survival of cells when challenged with ROS, enzymes for the reduction of lipid peroxides in *Borrelia* need to be identified.

Interestingly, *Borrelia* cells grown aerobically showed signs of membrane damage similar to those observed in cells exposed to various oxidants. The amount of MDA in untreated aerobically grown cells was equivalent to that observed in treated cells (Fig. 3). Also, EM indicated that cells grown microaerobically or aerobically had significantly more membrane damage than cells grown anaerobically (Fig. 6). This indicated that *Borrelia* membranes can be damaged simply by exposure to physiologically relevant concentrations of dissolved oxygen. In exponentially growing *E. coli*, Imlay and Fridovich (1991) have shown that both O_2_^-^ and H_2_O_2_ are generated by the autoxidation of components of the respiratory chain during oxygen metabolism. In contrast, *B. burgdorferi* is very metabolically limited and has no enzymes for the TCA cycle or respiration. Therefore, it seems more likely that sources of ROS are exogenous (e.g. innate immune response in the mammalian host) rather than endogenous. In addition, the current practice of growing *B. burgdorferi* under atmospheric oxygen could itself be unintentionally compromising cell integrity during *in vitro* manipulations.

Analyses of the *B. burgdorferi* genome indicates that only a few genes encoding putative oxidative stress/intracellular redox proteins (SodA, NapA, BosR, CoADR, Trx and TrxR) (Fraser *et al*., 1997) are present, compared with other bacterial pathogens, including other pathogenic spirochetes (e.g. *Treponema*, *Leptospira*). Despite this apparent ‘deficiency’ in the number of ROS-protective enzymes, *B. burgdorferi* cells appear to be able to cope with physiologically relevant levels of ROS. There are several factors that would contribute to this phenomenon: (i) as *B. burgdorferi* does not harbour the genes encoding respiratory enzymes nor metabolize oxygen, it seems unlikely that significant levels of ROS are generated via the incomplete reduction of O_2_ during cellular metabolism. This would suggest that potential ROS challenges to *B. burgdorferi* would come almost completely from extracellular sources with little contribution from intracellularly generated ROS; (ii) owing to a lack of understanding of the physiological conditions in vector and host tissues infected with *B. burgdorferi*, it is difficult to assess the levels and/or sites of the potential ROS challenge during the infective cycle and (iii) most importantly, analyses of the potential targets for ROS in *B. burgdorferi* strongly suggested that the major targets of oxidative damage are different and perhaps less extensive in this bacterium than in other bacterial pathogens (e.g. *E. coli*). Taken together, this suggests that *B. burgdorferi* would be innately more resistant to ROS and require a less extensive repertoire of enzymes to protect the cells from oxidative damage.

## Experimental procedures

### Strains, growth conditions and reagents

*Borrelia burgdorferi* strain B31A3 and strain B314 (Sadziene *et al*., 1993) were grown in modified BSK-II (Barbour, 1984) medium at 34°C under an atmosphere of 0–20% O_2_ with 5% CO_2_ and the balance N_2_. Cells density was determined using a dark-field microscope. All reagents were purchased from Sigma Chemicals, St. Louis, MO unless stated otherwise. *E. coli* strain Top10, strain TA4315 (*ahpCF*) (Storz *et al*., 1989) and CM1319 (*mutM*) (Bridges *et al*., 1996) were grown in Luria–Bertani (LB) at 37°C with shaking.

### Per cent survivability assays

*Borrelia burgdorferi* strain B31A3 was grown to a cell density of 5 × 10^7^ cells ml^−1^ in BSK II under microaerobic (3% O_2_) conditions, the culture split and the cells treated with varying concentrations of oxidants (0–50 mM *t*-butyl hydroperoxide or H_2_O_2_) at 34°C for 4 h. After the incubation, cells were diluted in fresh BSK II, plated on BSK plates and incubated 7–14 days at 34°C. Per cent survivability was calculated as the number of colonies on the treated plates versus the number of colonies on the untreated plates.

### Determination of the spontaneous mutation rate

To determine spontaneous resistance to coumermycin A1, *B. burgdorferi* B31A3 cells were grown under microaerobic conditions to a cell density of 5 × 10^7^ cells ml^−1^ and treated with 5 mM H_2_O_2_ for 1 h at 34°C. The cells were plated on BSK plates containing 0 or 250 ng ml^−1^ coumermycin A1 and incubated 7–14 days at 34°C. The resistance frequency was calculated as the number of colonies that are Cou^R^ per total number of cells plated.

### Measurement of DNA base lesions

*Borrelia burgdorferi* B31A3 cells were grown in BSK-II under microaerobic conditions to a cell density of 5 × 10^7^ cells ml^−1^, treated with various oxidants (1 or 10 mM t-butyl hydroperoxide, 1 or 10 mM H_2_O_2_, 10 mM paraquat or lipoxidase) for 4 h and total DNA was isolated using Wizard Genomic DNA Purification Kit (Promega Corp., Madison, WI). The number of base lesions was determined using the DNA Damage Quantification Colorimetric Assay kit (Oxford Biomedical Research, Oxford, MI) following the manufacturer's protocol. Briefly, 500 ng of DNA was mixed with an equal volume of 10 mM biotinylated aldehyde reactive probe (ARP) reagent and incubated for 1 h at 37°C. The DNA-ARP product was ethanol-precipitated using glycogen as a carrier, washed three times with 70% ethanol and resuspended in Tris-EDTA to give a final concentration of 0.5 µg ml^−1^. The DNA-ARP product was allowed to bind to the wells of 96-well microplate overnight at 37°C. After the binding, the wells were washed four times with TPBS (137 mM NaCl, 2.7 mM KCl, 10 mM Na_3_HPO_4,_ 2 mM KH_2_PO_4_, 0.5% Tween 20, pH 7.4). The HRP-streptavidin conjugate was diluted to 0.5 µg ml^−1^ in Assay Buffer (0.15 M NaCl, 10 mM Na_s_HPO_4_, 1.5 mM KH_2_PO_4_, 2.5 mM KCl, 5 mg ml^−1^ BSA, 0.1% Tween, pH 7.5), 100 µl was added to each well and the plate incubated at 100 r.p.m. for 1 h at room temperature (RT). After incubation, the wells were washed four times with TPBS, 100 µl of substrate was added to each well and incubated for 1 h at 37°C. The reaction was then quenched with 100 µl of 1 M sulphuric acid and the reaction was monitored at 450 nm. The number of aldehyde reactive probe (DNA base lesions) per 10^5^ bp DNA was determined using a standard curve. As a control, *E. coli* TA4315 cells were grown in minimal media to OD_600_ of 0.4, treated with 0 or 100 µM H_2_O_2_ for 30 min and DNA isolated. The number of base lesions was determined as described above. *B. burgdorferi* strain B314 cells were grown under microaerobic conditions and DNA isolated. The number of DNA lesions was determined as described.

To determine the amount of 8-oxoguanine in *B. burgdorferi* DNA, cells were grown and treated as described above and DNA isolated. The DNA was converted to single-stranded DNA by boiling the sample for 5 min followed by rapid chill on ice. The DNA was then digested with nuclease P1 for 2 h and then treated with alkaline phosphatase for 1 h following manufacturer's protocols. The resultant mixture was then centrifuged for 5 min at 6000 *g* and the supernatant used for the 8-oxoguanine ELISA assay (Oxford Biomedical Research, Oxford, MI). As a control, *E. coli* CM1319 (*mutM*) cells were grown in LB to OD_600_ of 0.4, treated with 0 or 100 µM H_2_O_2_ for 1 h and DNA isolated. The concentration of 8-oxoguanine was determined as above.

### Lipid analyses

To determine fatty acid content in *B. burgdorferi* total membranes, *B. burgdorferi* strain B31A3 was grown under anaerobic conditions to a cell density of 5 × 10^7^ cells ml^−1^, harvested by centrifugation (5000 *g*, 15 min, 4°C) and washed two times with HN (20 mM NaCl, 50 mM Hepes, pH 7.6) buffer. Cell pellets were analysed for fatty acid composition by fatty acid methyl ester (FAME) gas chromatography (Lipid Technologies, Austin, MN) and results are reported as percentage of total fatty acid content. To determine the effects of oxidants on fatty acid composition, a 1.5 l culture of *B. burgdorferi* strain B31A3 was grown under microaerobic conditions to a cell density of 5 × 10^7^ cells ml^−1^. The culture was split into 500 ml aliquots, the first was treated with 1 mM *t*-butyl hydroperoxide, the second with 0.25 mg of lipoxidase (17 700 units) and the third was untreated. All cultures were incubated for 12 h at 34°C. Cells were harvested by centrifugation (5000 *g*, 4°C, 15 min) and washed three times with HN buffer. The fatty acids present in the cell pellets were analysed by FAME gas chromatography (Industrial Laboratory, Wheat Ridge, CO). Fatty acids were reported as percentage of total cell mass.

### Measurement of MDA

*Borrelia burgdorferi* strain B31A3 was grown aerobically, microaerobically and anaerobically as described above to a cell density of 5 × 10^7^ cells ml^−1^. The microaerobic culture was split and treated with either 5 mM AAPH [2,2′-azobis(2-methylpropionamidine) dihydrochloride] or 250 mg of lipoxidase for 4 h at 34°C. To measure the amount of MDA, the cells were derivatized with thiobarbituric acid and analysed by HPLC as described by Seljeskog *et al*. (2006). After the incubation, all cells were harvested by centrifugation (1000 *g*, 5 min, 4°C) and washed three times with HN buffer. Each sample was resuspended in 50 µl of HN, mixed with 150 µl of 0.1 N perchloric acid, 150 µl of 40 mM thiobarbituric acid and 35 µl of 20% SDS, vortexed and heated at 97°C for 60 min. After cooling at −20°C for 20 min, 300 µl of methanol and 100 µl of 20% trichloroacetic acid was added and the samples were mixed vigorously and centrifuged (13 000 *g*, 6 min). The samples (10 µl) were then analysed with an Agilent Technologies 1200 series HPLC system using a C18 4.6 × 150 mm column with mobile phase 72:17:11 (50 mM KPO4, pH 6.8 : methanol : acetonitrile). Absorbance was monitored at 532 nm. Pure MDA standards (0–10 µM) were prepared in methanol for comparison. As a negative control, *E. coli* Top10 cells were grown to OD_600_ of 0.4, treated with 0 or 5 mM AAPH for 30 min and MDA measured as above. As a positive control, mouse myeloma SP2/O cells were cultured with HYQ-CCM1 (HyClone) medium at 37°C in a humidified 5% CO_2_ atmosphere, treated with 1 mM AAPH at 37°C for 4 h (Chotimarkorn *et al*., 2005) and MDA measured as above.

### Identification of lipid damage using Diphenyl-1-pyrenylphosphine fluorescent stain

*Borrelia burgdorferi* strain B31A3 cells were grown microaerobically as described above until a cell density of 5 × 10^7^ cells ml^−1^ was obtained. The culture was divided into two equal aliquots and the cells treated with 5 mM AAPH for 4 h at 34°C. After the incubation, all cells were harvested by centrifugation (1000 *g*, 5 min, 4°C) and washed three times with HN buffer. To visualize *Borrelia* cells, the cells were stained with PKH26 Red Fluorescent Cell Linker Dye (Sigma Aldrich) following the manufacturer's protocol. To visualize the lipid hydroperoxides in the cell membrane, the cells were counterstained with DPPP (Cayman Chemicals, MI) (Okimoto *et al*., 2000). Briefly, after cells were stained with the Red Fluorescent dye, 2 ml of rabbit serum was added to stop the reaction and the mixture incubated for 1 min at RT. Next, 4 ml of HN buffer was added, the cells harvested by centrifugation (1000 *g*, 10 min, RT) and washed three times with HN buffer. The cells were then resuspended in 1 ml of HN buffer, incubated at 34°C for 5 min and 30 µl of 2.5 mM DPPP was added. The incubation was then continued at 34°C for 5 min in the dark. After incubation, the mixture was centrifuged (1000 *g*, 10 min, RT) and the cells washed three times with HN buffer. The cells were then resuspended in 100 µl HN buffer and observed by fluorescence microscopy with excitation wavelengths 551 nm (Red Fluorescent) and 351 nm (DPPP), and emission wavelengths 567 nm (Red Fluorescent) and 380 nm (DPPP). As a positive control, mouse myeloma SP2/O cells were cultured with HYQ-CCM1 (HyClone) medium at 37°C in a humidified 5% CO_2_ atmosphere, treated with 1 mM AAPH at 37°C for 4 h (Chotimarkorn *et al*., 2005) and stained as described above. As a negative control, *E. coli* Top10 cells were grown to OD_600_ of 0.4, treated with 0 or 5 mM AAPH for 30 min and stained as described above.

### Electron microscopy

*Borrelia burgdorferi* cells were grown under microaerobic or anaerobic conditions to a cell density of 5 × 10^7^ cells ml^−1^. The microaerobic cultures were split and treated with lipoxidase for 4 h at 34°C. Cells were harvested by centrifugation (5000 *g*, 10 min, 4°C), washed and resuspended in HBSS (Lonza Group Ltd, Switzerland). The cells were fixed with Karnovsky's phosphate for 5 min at RT adsorbed to Formvar/carbon-coated grids (Ted Pella, Redding, CA) for 5 min and washed three times in H_2_O. The grids were stained with 1% ammonium molybdate and allowed to air-dry. Samples were examined using a Hitachi H7500 electron microscope (Hitachi High Technologies America, Pleasanton, CA).
